# Realizing serine/threonine ligation: scope and limitations and mechanistic implication thereof

**DOI:** 10.3389/fchem.2014.00028

**Published:** 2014-05-20

**Authors:** Clarence T. T. Wong, Tianlu Li, Hiu Yung Lam, Yinfeng Zhang, Xuechen Li

**Affiliations:** ^1^Department of Chemistry, The University of Hong KongHong Kong, China; ^2^State Key Laboratory of Synthetic Chemistry, University of Hong KongHong Kong, China; ^3^Shenzhen Institute of Research and Innovation of The University of Hong KongShenzhen, China

**Keywords:** peptide ligation, solid phase peptide synthesis, protein synthesis, chemoselectivity, peptide synthesis

## Abstract

Serine/Threonine ligation (STL) has emerged as an alternative tool for protein chemical synthesis, bioconjugations as well as macrocyclization of peptides of various sizes. Owning to the high abundance of Ser/Thr residues in natural peptides and proteins, STL is expected to find a wide range of applications in chemical biology research. Herein, we have fully investigated the compatibility of the STL strategy for X-Ser/Thr ligation sites, where X is any of the 20 naturally occurring amino acids. Our studies have shown that 17 amino acids are suitable for ligation, while Asp, Glu, and Lys are not compatible. Among the working 17 C-terminal amino acids, the retarded reaction resulted from the bulky β-branched amino acid (Thr, Val, and Ile) is not seen under the current ligation condition. We have also investigated the chemoselectivity involving the amino group of the internal lysine which may compete with the N-terminal Ser/Thr for reaction with the C-terminal salicylaldehyde (SAL) ester aldehyde group. The result suggested that the free internal amino group does not adversely slow down the ligation rate.

## Introduction

Chemical methods enabling merger of two fully side chain unprotected peptide segments at the termini, termed peptide ligation, provide access to complex, long peptides and even proteins beyond the limit of solid phase peptide synthesis (SPPS) (Kent, 2009). Numerous methodologies have emerged to be capable of delivering on specific peptide/protein targets. In particular, ever since the introduction of native chemical ligation (NCL) (Dawson et al., 1994; Tam et al., 1995; Dawson and Kent, 2000), protein chemical synthesis has become reachable and thus provided a general strategy for the flexible and precise incorporation of natural or unnatural elements into a protein molecule. The realization of NCL lies in the super nucleophilicity of the N-terminal cysteine to mediate a chemoselective peptide ligation, thereby enabling merger of two side chain unprotected peptide segments with the generation of the natural Xaa-Cys at the ligation site. Over the past two decades, many advances in the development of strategies and methods to improve and expand the NCL at other amino acids sites (Hackenberger and Schwarzer, 2008; Hemantha et al., 2012; Monbaliu and Katritzky, 2012; Verzele and Madder, 2013). Of particular importance, the success of NCL-desulfurization to realize the peptide ligation at the alanine site (Yan and Dawson, 2001; Wan and Danishefsky, 2007) has initiated the development of NCL at other amine acid sites using synthetic cysteine/selenocysteine surrogates (Canne et al., 1996; Wong et al., 2013c). Examples include the use of ß-mercaptophenylalanine (Crich and Banerjee, 2006), γ-mercaptovaline/penicillamine (Chen et al., 2008; Haase et al., 2008), ß-mercaptoleucine (Harpaz et al., 2010; Tan et al., 2010), γ-mercaptothreonine (Chen et al., 2010), δ-mercaptolysine (Ajish Kumar et al., 2009a; Yang et al., 2009), γ-mercaptoproline (Ding et al., 2011), ß-mercaptoglutamine (Siman et al., 2012), ß-mercaptoaspartate (Guan et al., 2013; Thompson et al., 2013), ß-selenol-phenylalanine (Malins and Payne, 2012), and selenol-proline (Townsend et al., 2012) to generates the ligation sites with Phe, Val, Leu, Thr, Lys, Pro, Gln, and Asp, respectively, by different research groups. Nevertheless, most of these β/γ mercapto unnatural amino acids are not commercially available and require tedious organic synthesis (Wong et al., 2013c). In addition, the usage of unique reacting functional groups at the ligating C-terminus and N-terminus, including thioester containing a phosphine group-azide and the α–ketoacid-hydroxylamine, has led to the thiol-independent ligation, Staudinger ligation (Nilsson et al., 2000) and KAHA ligation (Bode et al., 2006; Pattabiraman et al., 2012), respectively.

**Figure 1:**
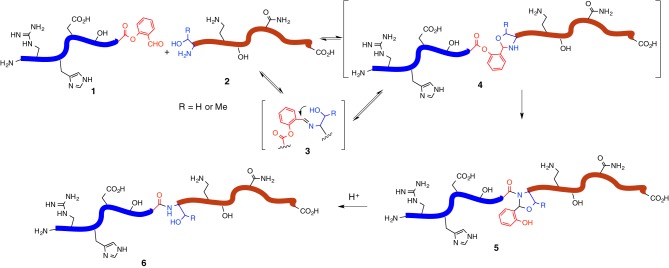
**Scheme of Ser/Thr ligation**.

In pursuing the development of the easy-handling chemoselective peptide ligation, we have recently introduced a serine/threonine ligation (STL) strategy, enabling the peptide ligation of side chain unprotected peptide segments with the generation of natural peptidic bond (Xaa-Ser/Thr) at the ligation site (Li et al., 2010). Although the ligations which could generate Ser/Thr at the ligation site have been developed by different groups (Okamoto and Kajihara, 2008; Ajish Kumar et al., 2009b; Thomas and Payne, 2009; Chen et al., 2010), our ligation strategy directly uses the natural serine or threonine residue at the N-terminus to mediate peptide ligation. This ligation strategy involves the merger of a peptide salicylaldehyde (SAL) ester and a peptide with N-terminal serine or threonine, to afford an *N,O*-benzylidene acetal at the conjugation site, followed by simple acidolysis to release the natural peptidic bond. Along the pathway of this ligation, the peptide SAL ester (SE) **1** would react with the amino group of the N-terminal serine or threonine residue of the second peptide segment **2** to give an imine species (*c.f.* **3**) reversibly, followed by the 5-endo-trig cyclization from the β-hydroxyl group of the N-terminal serine or threonine residue to afford an oxazolidine (*c.f.* **4**). Then an irreversible *O* → *N* 1,5 acyl transfer would afford a stable amide intermediate (*c.f.* **5**). Next, the resultant *N,O*-benzylidene acetal could be readily removed via acidolysis to restore the natural Xaa-Ser/Thr peptidic linkage at the ligation site (*c.f.* **6**) (Figure 1).

We have successfully applied this method to convergently synthesize various peptides/proteins including MUC1 glycopeptide and an enzyme acylphosphatase (Xu et al., 2013; Zhang et al., 2013). In addition, STL-mediated peptide cyclization has been used to synthesize daptomycin and other cyclic peptides of various sizes (Lam et al., 2013; Wong et al., 2013a,b; Zhao et al., 2013). More recently, STL has been used in the form of expression protein ligation, producing a RNase-peptiod conjugate (Levine et al., 2014). In this article, we wish to address several issues around this ligation. First, we feel necessary that the effect of the unprotected lysine on the chemoselectivity needs to be carefully studied. Secondly, we aim to study the scope and limitations of STL with different C-terminal amino acid residues.

## Materials and methods

### Instruments

All commercial materials (Aldrich, Chemimpex, and GL Biochem) were used without further purification. All solvents were reagent grade or HPLC grade (RCI or DUKSAN). Anhydrous tetrahydrofuran (THF) was freshly distilled from sodium and benzophenone. Dry dichloromethane (CH_2_Cl_2_) was distilled from calcium hydride (CaH_2_). All separations involved a mobile phase of 0.1% TFA (*v/v*) in acetonitrile (solvent A) and 0.1% TFA (*v/v*) in water (Solvent B). HPLC separations were performed with a Waters HPLC system equipped with a photodiode array detector (Waters 2996) using a Vydac 218TP™ C18 column (5 μm, 300 Å, 4.6 × 250 mm) at a flow rate of 0.6 mL/min for analytical HPLC and XBridge™ Prep C18 OBD™ column (10 μm, 300 Å, 30 × 250 mm) at a flow rate of 15 mL/min for preparative HPLC. Low-resolution mass spectral analyses were performed with a Waters 3100 mass spectrometer.

### Solid phase peptide synthesis (Boc chemistry)

The following Boc amino acids were purchased from GL Biochem and Chemimpex and used in the solid phase synthesis: Boc-Ala-OH, Boc-Gly-OH, Boc-Ile-OH, Boc-Phe-OH, Boc-Lys(2-Cl-Z)-OH, Boc-Phe-OH, Boc-Ser(Bzl)-OH, Boc-Thr(Bzl)-OH, Boc-Tyr(Bzl)-OH, Boc-Val-OH, Boc-Pro-OH. Based on the SAL linker synthesized previously (Wong et al., 2013b), the standard Boc-SPPS protocol was employed. A solution of 20% piperidine in DMF (10 mL) was added to the loaded resin (0.5 mmol/g) and the mixture was gently agitated for 1 h to remove the acetyl group. The solvent was drained and the resin was washed with DMF (10 mL × 3). A solution of the first amino acid to be coupled (4.0 equiv. relative to resin capacity), PyBOP (4.0 equiv.) and DIPEA (8.0 equiv.) in anhydrous DMF (10 mL) was added. The mixture was gently agitated for 3 h. The Boc group was removed with neat TFA (2 × 5 min) followed by sequential washing with CH_2_Cl_2_ (10 mL × 3), DMF (10 mL × 3), and CH_2_Cl_2_ (10 mL × 3). A solution of Boc-Xaa-OH (2.0 equiv. relative to resin capacity), HATU (2.0 equiv.), and DIPEA (4.0 equiv.) in anhydrous DMF (10 mL) was added to the resin. The mixture was gently agitated for 45 min. The coupling was repeated followed by sequential washing with DMF (10 mL × 3) and CH_2_Cl_2_ (10 mL × 3). For global deprotection, a mixture of TFMSA/TFA/thioanisole (1:8.5:0.5, *v/v/v*) was added to the resin bound peptide at 0°C and the mixture was gently agitated for 1 h. The resin was then washed with CH_2_Cl_2_ (10 mL × 6). Analytical RP-HPLC with gradient 5–90% ACN containing 0.1% TFA over 20 min was used to confirm the purity of the peptides.

### Ozonolysis

The resin bound peptide was swollen in CH_2_Cl_2_/TFA (95:5, *v/v*) at −78°C. After the mixture was treated with O_3_ at −78°C for 10 min, Me_2_S (10.0 equiv. relative to resin capacity) was then added at −78°C. The reaction mixture was allowed to warm to room temperature over 30 min. The mixture was filtered and concentrated under *vacuo* and subjected to the HPLC purification. The peptides containing Cys, Met, and Trp are not compatible with the ozonolysis condition.

### Solid phase peptide synthesis (Fmoc chemistry)

Synthesis was performed manually on Rink amide-AM resin or 2-chlorotrityl chloride resin. Peptides were synthesized under standard Fmoc/t-Bu protocols. The deblock mixture was a mixture of 20/80 (vol/vol) of piperidine/DMF. The following Fmoc amino acids from GL Biochem were used: Fmoc-Ala-OH, Fmoc-Glu(OtBu)-OH, Fmoc-Gln(Trt)-OH, Fmoc- Gly-OH, Fmoc-Lys(Boc)-OH, Fmoc-Met-OH, Fmoc-Phe-OH, Fmoc-Trp(Boc)-OH, and Fmoc-Cys(StBu)-OH. Upon completion of synthesis, the peptide resin was subjected to a cleavage mixture. The resin was filtered and the combined filtrates were blown off under a stream of condensed air. The crude product was triturated with cold diethyl ether to give a white suspension, which was centrifuged and the ether subsequently decanted. The remaining solid was ready for HPLC purification. The peptide N-acyl-benzimidazolinone (Nbz) was prepared according to Blanco-Canosa and Dawson's protocol (Blanco-Canosa and Dawson, 2008). The on-resin phenolysis for the synthesis of peptide SE was conducted by adding Na2CO3 (10.0 equiv) and a solution of SAL dimethyl acetal (1.0 mL/0.1 g resin) in dry DCM/THF (1/3, vol/vol) to the resin-bound peptide Nbz. The suspension was stirred at room temperature for 16 h. The resin was then filtered and washed with DCM. The combined filtrates were concentrated under a stream of condensed air. The oily residue was treated with TFA/H_2_O (95/5, v/v) for 1 h. The solvent was blown off under a stream of condensed air. The crude product was triturated with cold diethyl ether to give a white suspension, which was centrifuged and the ether subsequently decanted. The remaining solid was ready for HPLC purification. Using this on-resin phenolysis approach, several esters were synthesized in 20–30% yield from the resin. Analytical RP-HPLC with gradient 5–90% ACN containing 0.1% TFA over 20 min was used to confirm the purity of the peptides.

### Ser/Thr ligation

The peptide SE and the N-terminal peptide were dissolved in pyridine/acetic acid (1:1 mole/mole) at a concentration of 1–5 mM at room temperature and were stirred for 2–20 h. The solvent was removed under *vacuo*. The crude ligated peptide was treated with 95% TFA for 10 min to remove the O, *N*-benzylidene acetal. The solvent was then blown off by a stream of air and the residue was purified by Prep-HPLC system with 15–80% ACN with 0.1% TFA in 45 min.

## Results

### Effect of the side-chain unprotected lysine

STL involves the imine formation as the first capture step, from the aldehyde group of the C-terminal SE and the amino group of the N-terminal serine or threonine. Indeed, the free amino groups of the internal Lys is likely to compete with the amine of the N-terminal of Thr/Ser residue to form the imine with the C-terminal SE aldehyde. Thus, it raises a question whether the presence of Lys would slow down or even inhibit the ligation. In this regards, we designed a competitive experiment to study the effect of Lys at the different positions on the ligation efficiency. We have prepared an N-terminal peptide without any Lys residue (K0) and a series of N-terminal peptide with Lys residue at different position (K2–K5) (Table 1). We intended to compare the ligation rate of the Lys-containing peptide relative to the K0 peptide, when reacting with the same peptide SE.

**Table 1:** **Sequence of model peptides used in competitive experiment**.

**Name**	**Sequence**	**Molecular Weight**
SAL-ester (SE)	NH_2_-AIFPNPF-SAL ester	908.44
K0	NH_2_-SVAFGA-CO_2_H	550.28
K2	NH_2_-S**K**AFGA-CO_2_H	579.30
K3	NH_2_-SV**K**FGA-CO_2_H	607.33
K4	NH_2_-SVA**K**GA-CO_2_H	531.30
K5	NH_2_-SVAF**K**A-CO_2_H	621.35

Specific the position of Lys (K).

**Figure 2:**
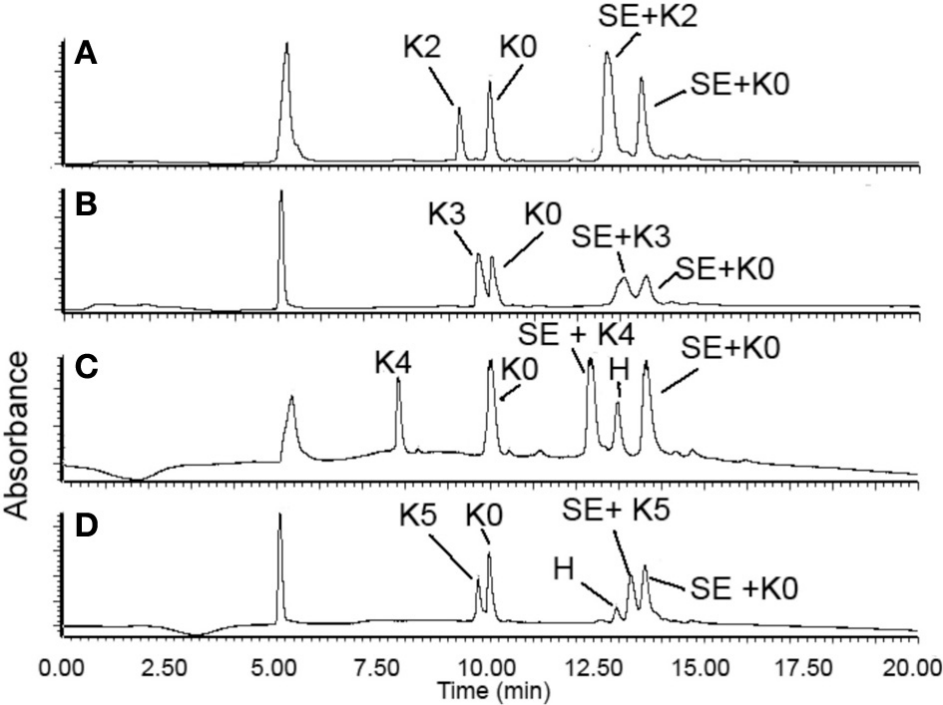
**RP-HPLC spectra of STL of the peptide SAL ester with peptides K2–K5**. SE = peptide-SAL ester; H = hydrolysis of peptide-SAL ester; SE+K_*X*_ = ligation product. Ligation time: **(A)** (1.5 h) and **(B–D)** (4 h).

In each ligation, we dissolved 1.0 equiv. of peptide SE and 1.0 equiv. of K0 peptide and 1.0 equiv. of any peptide from K2–K5 peptides in the ligation buffer at the concentration of 5 mM. The crude reaction mixture was analyzed by LC-MS and the UV peaks of the ligation products were integrated and compared. In this setting, peptide SE was fully consumed and the N-terminal peptides (K0 and K2–5) were in excess. Thus, the ratio of the UV integral could be correlated with the relative reaction rate. As seen from Figure 2, the K3–K5 reacted very similarly to the K0 peptide, with roughly equal integrals, and the ligations were completed in 4 h. These results have indicated that the STL was not significantly affected by the presence of Lys in the sequence, as the K3–5 peptides were having a similar conversion to ligation products as K0. Thus, we can concluded that, although the Lys would react with the peptide SE, the nonproductive imine formation is reversible and rapid, thus not affecting the ligation pathway.

Interestingly, K2 behaved differently from K3–K5 peptides. First, the reaction was rapid with completion in 1.5 h, in contrast with 4 h for K3–K5 ligations. Secondly, as determined from the LC spectrum, the K2 peptide reacted much faster (~2× faster) than K0 peptide (Figure 2A). The leftover of K2 was much less than K0, which excluded that possibility that more K2 was accidently added than K0 peptide to the reaction to cause the more K2 ligated product. This indicated that the second position (next to N-terminal Ser/Thr) could enhance the ligation.

### Effect of the ligating amine acids

The C-terminal amino acid next to the ligation site has significantly affected the efficiency of the NCL. For instance, the sterically hindered β-branched amino acids, including Thr, Val, Ile, resulted in the incomplete ligation after 48 h (Hackeng et al., 1999). The ligation with the C-terminal proline was also very slow, which is proposed to result from the reduced electrophilicty imposed by the n->π^*^ orbital interaction (Pollock and Kent, 2011). Analogously, we aimed to systematically evaluate the compatibility and reactivity of all 20 amino acid at the C-terminus of the peptide SE. We elected two peptides: NH_2_-AEGSQAKFG**X**- SE as the C-terminal peptide where X represents different amino acids, and NH_2_-SPKALTFG-CO_2_H as the model peptide containing Ser at the N-terminus. The necessary peptide SAL esters (X = A, G, T, S, V, L, I, P, F, Y, D, E, N, Q, H, and K) were prepared by Boc-SPPS (Wong et al., 2013b), while the peptide SAL esters (X = W, C, M, and R) were synthesized via Fmoc-SPPS (Zhang et al., 2013) due to the incompatibility of ozonolysis (M, C, and W) and TFMSA deprotection of the tosyl group of Arg. The ligation of two unprotected peptide segments was performed as follows: two peptide segments were dissolved in pyridine acetate buffer (1:1, mole: mole) at the concentration of 1 mM at room temperature. The reaction process was monitored by analytical LC-MS at different time points (2, 6, and 15 h). The conversions of all ligations were determined after 2 h were summarized in Table 2.

**Table 2:** **Conversion percentage of different C-terminal amino acid-SAL-ester at 2 h**.

**Entry**	**C-terminal a.a**.	**Conversion (%)**
1	Ala	87.1
2	Gly	85.9
3	Ser	84.8
4	Gln	78.7
5	Thr	71.3
6	Phe	67.7
7	Cys(StBu)	65.2
8	Val	45.5
9	Ile	41.8
10	Met	38.5
11	Asn	38.5
12	Tyr	33.7
13	Leu	33.4
14	His	28.6
15	Trp	24.8
16	Arg	20.5
17	Pro	7.9
18	Asp	–
19	Glu	–
20	Lys	–

As seen in Table 2, Ala, Gly, Ser, Glu, Thr, Phe, and Cys(StBu) appeared to have the fastest conversions, in which >60% of the peptide ester was consumed at 2 h and the ligation was completed within 6 h. Interestingly, the β-branched amino acid Thr undergoes at a similar conversion of ligation to the least hindered amino acid glycine. Val, Leu, Ile, Asn, Phe, Met, and Tyr represent the second batch of amino acid with >30% conversions in the first 2 h and reaction was completed within 6 h. Then the conversion of Arg, Trp, Pro, and His ranged from 7.9 to 28.6% showed to be the slowest. In particular, the ligation with C-Pro and Arg could not be completed after 15 h. In addition, we found that the C-terminal Asp, Glu, and Lys were not compatible with the current STL condition, due to the instability of the peptide SE when the first amino acid contains nucleophilic side-chain functionalities.

### Acidolysis

**Figure 3:**
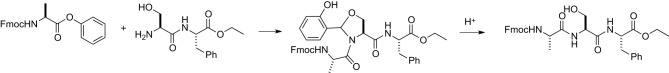
**Acidolysis of N,O-benzylidene acetal intermediate**.

The second step of this STL involves acidolysis to release the *N,O*-benzylidene acetal generated at the conjunction site (Figure 3). Thus, we performed various acidolysis conditions. We have prepared a tripeptide containing the *N,O*-benzylidene acetal and treated it with a range of acidic conditions, including 95, 50, 10, 5, and 1% TFA. The reaction progresses were monitored by LC-MS (Table 3).

**Table 3:** **Summary of the LC-MS analysis of the reaction progress**.

**Entry**	**Conditions**	**Conversion (%)**		
		**5 min**	**30 min**	**4 h**
1	95% TFA in H_2_O	100	–	–
2	50% TFA in H_2_O	100	–	–
3	10% TFA in H_2_O/CH_3_CN (1:1, v/v)	–	63	100
4	5% TFA in AcOH/H_2_O (1:1, v/v)	–	85	100
5	1% TFA in AcOH/H_2_O (1:1, v/v)	–	20	67
6	5% TFA in H_2_O/CH_3_CN (1:1, v/v)	–	40	100
7	1% TFA in H_2_O/CH_3_CN (1:1, v/v)	–	7	42

As seen in Table 3, 50% TFA and above can rapidly remove the benzylidene acetal within 5 min. The acidolysis with 5–10% TFA could be completed within 4 h. When 1% TFA was used, the acidolysis became very slow.

## Discussion

**Figure 4:**
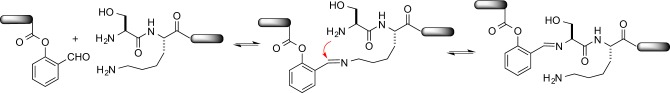
**Proposed mechanism to explain the accelerated rate of K2 peptide**.

STL has emerged as a new tool to deliver synthetic peptides/proteins with a natural peptidic linkage via the convergent ligation of two side chain unprotected peptide segments. This method uses the natural amino acid, the N-terminal serine or threonine, to mediate a chemoselective peptide ligation, thus featuring operational simplicity. Herein, we have fully investigated the effect of unprotected Lys on the ligation. In our proposed mechanism of STL, the amino group of the N-terminal Ser/Thr was first condensed with the aldehyde to form an imine. Thus, it makes one wonder whether the amino groups of the internal Lys in the peptide sequence could slow down the ligation by competing with the amino group of the N-terminal Ser/Thr. We have conducted competitive experiments and concluded that the reaction rate between an N-terminal peptide without Lys (K0) and the N-terminal peptides with one Lys at different position including K3–K5 was not significant. Interestingly, it is worthy to note that K2 peptide with a Lys next to the N-terminal serine showed an accelerated reaction rate relative to K0. To explain this result, we propose that the Lys acts to help capture and deliver the C-terminal peptide SE to the N-terminal Ser residue via an 11-membered ring transition state via proximity-induced imine exchange (Kovaříček and Lehn, 2012), thus benefitting the ligation with the K2 peptide (Figure 4).

Another important issue that we have addressed was the compatibility and the relative conversion percentage of the C-terminal amino acid of the peptide SE under the STL conditions. In the NCL-mediate peptide ligation, Dawson have used LYRAX-SR and CRANK as the model peptides at 37°C to study the relative conversion percentage of all C-terminal amino acids (Hackeng et al., 1999). Their results have led them to classify the C-terminal amino acids into four groups: fast reaction with His, Gly, and Cys (≤4 h), modest reaction with Phe, Met, Tyr, Ala, and Trp (≤9 h), slow reaction with Asn, Ser, Gln, Lys, Arg (≤24 h), and very slow reaction with Leu, Thr, Val, Ile, and Pro (≥48). The compatibility of C-terminal Asn, Gln, Asp, and Glu with NCL were controversial, as significant levels of β- and γ– linked byproducts during ligation were observed by others (Camarero and Muir, 2001; Villain et al., 2003).

Our similar studies, but with NH_2_-AEGSQAKFG**X**- SE and NH_2_-SPKALTFG-CO_2_H as model peptides ligating at room temperature, have indicated that the conversion rate of C-terminal amino acids under STL conditions does not follow the same trend as that of NCL. First, the hindered β-branched amino acids (e.g., Val, Thr, Ile) do not retard the formation of product significantly. Indeed, the ligation with C-terminal threonine is very fast, similarly to Ser, Gln, Gly, and Ala. Secondly, the less favorable choices for STL are Arg, Trp, Pro, and His. The factors causing the slow reaction at these reactions sites are not clear to us yet. Thirdly, the C-terminal Asn sometime poses a problem in NCL, due to the formation of C-terminal succinimides(Gross et al., 2005), while under the STL condition, the ligation with the peptide Asn SE proceeded smoothly without significant side products. Lastly, the peptide SE at Asp, Lys, and Glu were very unstable for the isolation, making the study of ligation impossible, thus they are not compatible with STL. In addition, we found that the peptide SE with the C-terminal cysteine generated unidentifiable side products during the STL. However, this problem could be easily solved using C-terminal Cys (S-*t*Bu), since the STL does not use reducing conditions.

Biomolecules obtained from chemical synthesis have been highlighted by their high homogeneity and flexible modifications, in contrast with biologically controlled methods, in particular for proteins with post-translational modifications. Thus, the development of the novel and efficient methods and strategies readily adoptable by the non-expertise researchers will be important in this context. We have recently developed a STL for convergent peptide synthesis and demonstrated that this method is capable of delivering synthetic peptides/proteins. This method features high chemoselectivity, operational simplicity and use of common regents. Our studies here have shown that, although STL involves imine capture step, the internal Lys does not interfere with the ligation pathway and indeed Lys next to the N-terminal Ser/Thr can enhance the ligation efficiency. In addition, 17 amino acids at the C-terminus are suitable for ligation, with Asp, Glu and Lys not compatible. Among working 17 C-terminal amino acids, the slow reaction resulted from the bulky β-branched amino acid in NCL were not observed under STL conditions. Nevertheless, the ligation with C-terminal Pro and Arg was very sluggish and thus not recommended for STL.

### Conflict of interest statement

The authors declare that the research was conducted in the absence of any commercial or financial relationships that could be construed as a potential conflict of interest.
